# Overcoming Perfusion Impairment in the Ischemic Brain: A Review

**DOI:** 10.3390/neurosci7040089

**Published:** 2026-08-14

**Authors:** Andrew T. McKenzie, Aschwin de Wolf, Alexander Grotemeyer, Emil Kendziorra, Alexander German

**Affiliations:** 1 Apex Neuroscience, Salem, OR 97317, USA; 2 Advanced Neural Biosciences, Portland, OR 97220, USA; aschwin@advancedneuralbio.com; 3 Department of Neurology, Paracelsus Medical University, Campus Nuremberg (PMU Nuremberg), Klinikum Nuremberg, 90471 Nuremberg, Germany; 4 European Biostasis Foundation, 8197 Rafz, Switzerland; 5 Department of Molecular Neurology, Universitätsklinikum Erlangen, Friedrich-Alexander-Universität Erlangen-Nürnberg, 91054 Erlangen, Germany

**Keywords:** brain banking, no-reflow phenomenon, mechanical perfusion, cerebral ischemia, colloid osmotic pressure

## Abstract

Perfusion impairment is one of the major barriers to using machine perfusion to prepare brain tissue for research and clinical applications. As ischemia progresses, multiple mechanisms, including intravascular obstructions, perivascular cellular edema, mural cell contraction, and vessel wall breakdown, progressively limit the uniform delivery of preservative solutions. This problem is particularly important for connectomics, wherein successful preservation requires both widespread distribution of preservative chemicals and the maintenance of the cellular ultrastructure needed for circuit reconstruction. We conducted a narrative review of interventions used to ameliorate perfusion impairment across multiple fields, including organ transplantation, resuscitation medicine, forensic pathology, embalming, and neuroscience. We evaluated the evidence for these approaches in their original contexts and considered their potential application for brain banking aimed at preserving tissue for connectome reconstruction. We classify interventions into several categories, including anticoagulants, fibrinolytics, vasodilators, washout solutions, surfactants, osmotic agents, colloids, hypothermia, and perfusion pressure optimization. Many of these interventions have the potential to improve perfusion, but each also carries tradeoffs that may adversely affect tissue preservation. As connectomics advances toward profiling larger volumes of human brain tissue, overcoming perfusion impairment is likely to become an increasingly important challenge. This review provides a mechanistic framework for evaluating existing interventions and guiding the development of future perfusion protocols.

## 1. Introduction

Perfusion, derived from the Latin perfundere (“to pour through”), is the passage of fluid through the vasculature of a tissue. In normal physiology, it usually refers to the delivery of blood to capillary beds, where oxygen, nutrients, and waste products are exchanged between blood and tissue. The artificial or mechanical perfusion of non-blood fluids through the vasculature is also widely used in neuroscience research, and is often done postmortem. For example, artificial perfusion is used in animal research [1], brain banking [2], anatomical research [3,4], neurosurgical education [5], tissue clearing [6], vascular corrosion casting [7], and forensic medicine [8]. Preparing high-quality brain tissue via perfusion fixation is especially important for connectomics, which aims to map the synaptic wiring of neural circuits, and is advancing toward mapping larger volumes of the mammalian brain [9].

The success of a perfusion procedure depends critically on achieving adequate distribution throughout the cerebrovascular system, which is challenging due to factors that accumulate during ischemia. Perfusion quality is progressively compromised in a process that begins within just minutes of ischemia [10,11]. As a result, the spatial distribution of perfusion in an ischemic brain can be patchy and unpredictable, with some regions of tissue that receive inadequate perfusion even when the perfusion achieved in other areas is better [11,12]. The loss of uniform fluid distribution through the vasculature is a phenomenon known as perfusion impairment.

The problem of perfusion impairment is not unique to brain tissue preparation. It has been addressed in several fields. For example, in organ transplantation, methods to improve perfusion are critical for expanding the pool of usable donor organs and improving outcomes [13]. In resuscitation medicine, restoring cerebral microcirculation after cardiac arrest is essential for neurological recovery [14]. In postmortem angiography, techniques to restore vascular patency enable detailed imaging of the cerebral vasculature [15]. In embalming, more than a century of practical experience in arterial embalming have led to the development of formulations and procedures designed to achieve thorough capillary flow and tissue penetration [16]. Finally, in basic neuroscience research, although perfusion impairment due to ischemia is usually not relevant, high-quality perfusion fixation is a key method used for preserving ultrastructure for electron microscopy [17].

Different fields assess perfusion quality in different ways. Histological evaluation of blood vessel clearance is generally considered the gold standard for assessing perfusion quality, though it is limited by the requirement for sampling [12]. Other approaches include macroscopic inspection, tracer-based measurements, neuroimaging, and, in the resuscitation literature, neurological recovery of the organism after cardiac arrest as an indirect proxy. Flow–pressure relationships measured during perfusion can also provide real-time information about the aggregate vascular resistance that is present.

An important concept is the no-reflow phenomenon. The no-reflow phenomenon commonly refers to the failure to restore adequate microvascular perfusion, despite a restoration of circulatory flow in larger vessels [10,18]. For example, one study subjected cats to 5, 15, and 30 min of cardiac arrest followed by successful resuscitation and 30 min of spontaneous recirculation [14]. Even after they restored circulation, no-reflow persisted in 7%, 30%, and 65% of the forebrain in these groups, respectively [14]. Although perfusion impairment is a broader term that also includes other mechanisms, such as vasogenic edema due to vessel wall compromise or obstructions to flow in large vessels, the no-reflow phenomenon is a key paradigm in investigating why perfusion worsens with increasing ischemia time. Notably, some data has suggested that no-reflow can be present but partially reversible in some cases, especially with shorter periods of ischemia [19].

To the best of our knowledge, there has not yet been a synthesis of the approaches that have been used to overcome perfusion impairment, and how they might be applied to the perfusion of the ischemic brain. Each field has developed its own literature, terminology, and practices, often in isolation from one another. As a result, potentially valuable insights from one domain may not have yet been translated to others.

Throughout this review, we frequently use the term “ischemic” rather than “postmortem” when describing the underlying pathophysiology, because the mechanisms of perfusion impairment are driven by the cessation of blood flow rather than by any particular clinical determination of death. In addition, much of the relevant literature comes from studies of ischemia in living subjects, such as animal models subjected to cardiac arrest prior to resuscitation.

Improving perfusion quality is thought to be especially useful for brain banks that aim to preserve tissue with sufficient ultrastructural integrity to allow the tracing of synaptic connections, which is needed for connectomic study [20,21]. This is because we know from research in animal models and human brains that perfusion is a useful approach to achieve uniform tissue preparation at the ultrastructural level across the entire brain [2,4,17,22]. In perfusion fixation, a large volume of fixative is considered important in order to achieve a high preservation quality, which requires good perfusability [23]. Depending on the preservation method and the tolerance of subsequent imaging methods, it is possible that even relatively modest regions of perfusion failure could render portions of a specimen unsuitable for connectomic reconstruction with current electron microscopy or expansion microscopy methods.

In this review, we aim to conduct an evaluation of the various methods used to overcome perfusion impairment across multiple fields. While it is not possible to identify all of the methods that have been proposed to address this problem, we aim to identify as many of the well-established methods as possible. We evaluate the evidence for their effectiveness in their original contexts and consider the mechanistic framework for understanding how these interventions work. We have a particular focus on whether they might be useful in brain banking workflows designed to preserve connectomic information via perfusion. However, we hope this review will be useful for researchers working to improve perfusion outcomes in brain tissue preparation across a wide range of fields.

## 2. Review Methods

This is a narrative review that synthesizes the literature on interventions for perfusion impairment across multiple fields. We searched the available literature using Google, PubMed, and Google Scholar through June 2026, without a lower date limit, using combinations of terms related to cerebral no-reflow, ischemic perfusion impairment, postmortem perfusion, machine perfusion, perfusion fixation, and the intervention classes discussed below. We supplemented these searches by reviewing the references and citing studies of key articles, such as [10]. Studies were included if they described methods to improve or restore perfusion in ex vivo tissues or organs, provided mechanistic information about perfusion impairment, or reported outcomes related to ischemic perfusion quality. Studies were excluded if they did not provide information relevant to the mechanisms, assessment, or mitigation of perfusion impairment. Manuscripts were selected iteratively, with priority given to studies directly involving the ischemic brain, while also including evidence from other organs and fields when it was mechanistically relevant to brain perfusion. Because this is a narrative rather than systematic review, the search and study-selection process was iterative and was not intended to be exhaustive. We organized the literature by intervention class. For each class, we attempted to develop a mechanistic framework for how each class addresses perfusion impairment, with particular attention to brain tissue applications.

## 3. Mechanisms of Perfusion Impairment

Perfusion impairment is caused by several interacting mechanisms, which begin within minutes of circulatory arrest and progressively worsen with increasing ischemia time [24]. We briefly discuss the four classes of mechanisms that, through our review of the existing literature, we have found to be the most important.

### 3.1. Intravascular Obstructions

Two types of intravascular obstructions are most commonly observed on gross examination of the ischemic brain: agonal thrombi and postmortem clots (also known as cruor) [25]. Agonal thrombi are frequently yellow colored on gross examination, and as a result they are also called “chicken fat clots”. They are more adherent to vessel walls, and only occur in cases of slow death (thus, “agonal”) [26]. Microscopically, agonal clots are made of a loose network of platelets and fibrin, with some areas of more condensed fibrin aggregates [27].

**Figure 1:**
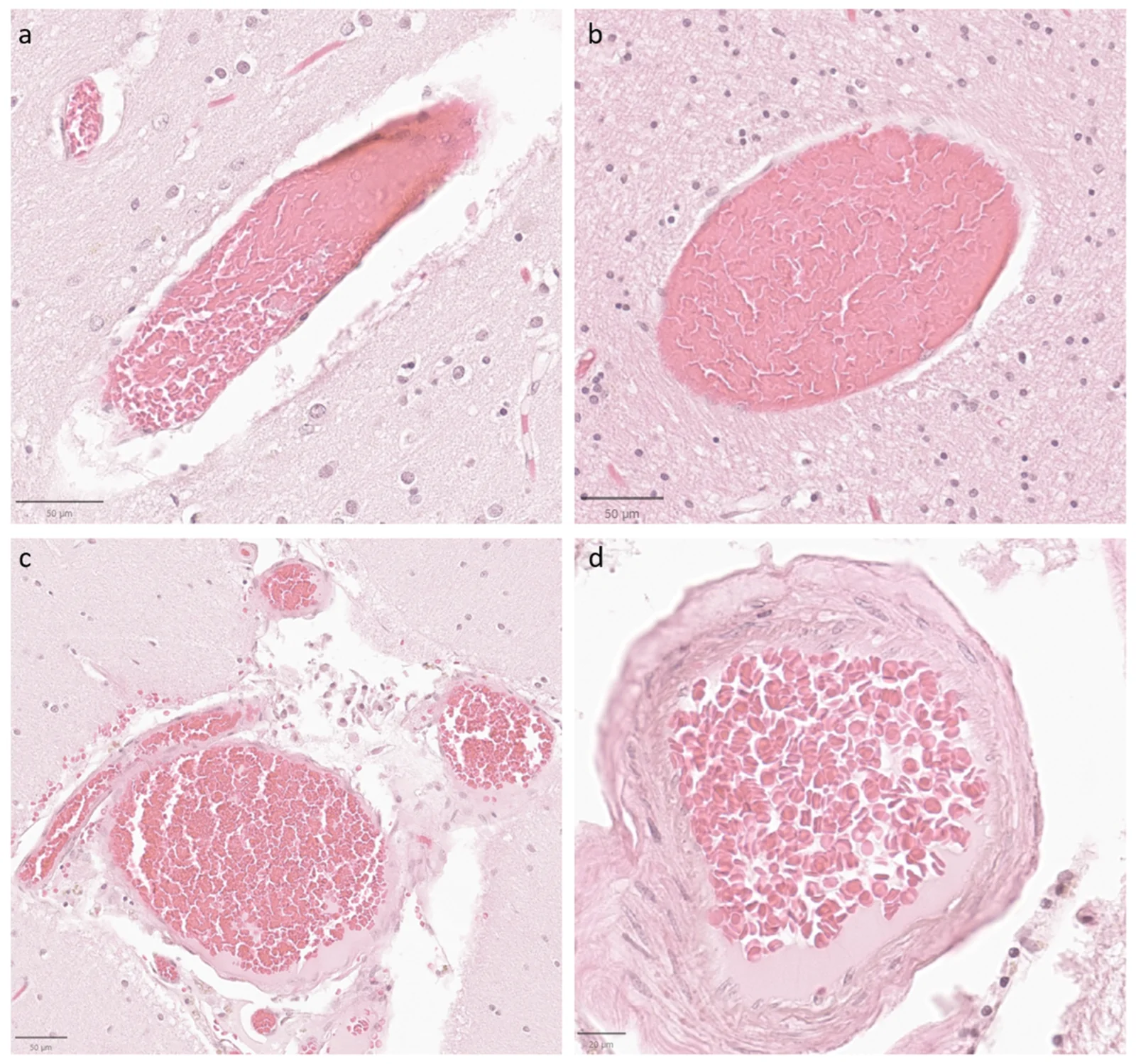
Examples of intravascular erythrocyte aggregation following prolonged ischemia. All images are taken from publicly available whole slide images previously described [12]. This is from Donor #166, an 83-year-old female donor with a postmortem interval of approximately 27 h, whose brain was immersion fixed in formalin and stained with hematoxylin and eosin. Erythrocyte aggregates consistent with rouleaux formation are visible within blood vessels across multiple brain regions. (**a**) Left frontal cortex. Note the gradient of erythrocyte packing density along the length of the vessel. (**b**) Left frontal cortex. (**c**) Left cerebellum. (**d**) Left temporal white matter. Scale bars: 50 µm (**a**–**c**), 20 µm (**d**).

Postmortem clots, on the other hand, are dark red or black, even more friable, and non-adherent to the vessel wall. They are found universally at autopsy regardless of the manner of death [26]. Mechanistically, postmortem clots form through passive erythrocyte aggregation (rouleaux formation) and gravitational sedimentation after circulatory arrest (Figure 1). Rouleaux formation is thought to be passive, reversible, and mediated by fibrinogen and other plasma proteins, as opposed to active coagulation [28,29]. Microscopically, postmortem clots consist primarily of red blood cells and a few scattered white blood cells, although strands and clumps of fibrin are also present in most of them [27].

Agonal thrombi and postmortem clots can coexist in the same vessel, and the boundary between them may not always be sharp, particularly on gross examination alone [25,26,30].

It is a long-reported finding that blood tends to be more liquid (i.e., have fewer clots) after sudden death, for example due to trauma [31,32]. Centuries after this was first reported, the mechanism for it is still unclear. One obvious contributing factor is that in sudden death it is expected that there would be no agonal thrombi, but this may not be the whole story [33].

In the focal cerebral ischemia literature, leukocytes (or less commonly platelets) have been visualized to clearly obstruct capillary-sized blood vessels, and this seems to be a significant contributor to the no reflow phenomenon after recanalization [34]. Likewise, in the heart, leukocyte depletion prior to focal myocardial ischemia has been shown to substantially decrease capillary plugging and reduce vascular resistance during reperfusion [35]. However, this mechanism is much less well established in the brain after global cerebral ischemia. For example, one study found that cyclophosphamide-induced leukopenia prior to 30 min of global cerebral ischemia did not reduce the severity of no-reflow in the gerbil brain during blood reperfusion [36]. Microscopically, white blood cells have also not been found to frequently block vessels after global forebrain ischemia [37]. Therefore, the effect of leukocyte plugging on perfusion impairment during brain banking is uncertain, and to the best of our knowledge, it has not yet been clearly demonstrated to play a major role after global cerebral ischemia. If it were found to be a significant factor, it would suggest that perfusion impairment could be lower in brain donors with leukopenia.

Another potential category of intravascular obstruction is capillary microthrombi—i.e., small thrombi that form within capillaries during the agonal or early ischemic period. A priori, there is reason to expect that microthrombi would form in capillaries after circulatory arrest, as blood stasis creates conditions favorable for coagulation. However, microscopy studies examining brain vasculature after cardiac arrest have not found significant thrombi (although notably, the evidence either way is not extensive) [38,39,40]. Despite this, premortem or peri-arrest heparin and fibrinolytics have been found in some studies to ameliorate perfusion impairment [41,42,43]. This suggests that either (a) capillary microthrombi are in fact present and meaningfully affect perfusate flow, but are sparse enough to be missed in microscopy of sectioned tissue or (b) the benefit of these pharmacologic agents may come not from preventing capillary microthrombi per se but from making agonal thrombi or recently formed postmortem clots less extensive or stable.

### 3.2. Perivascular Cellular Edema

Perivascular cellular edema occurs within minutes of ischemia. The failure of ATP-dependent ion pumps causes extracellular water, which is particularly abundant within the vasculature in the form of plasma, to influx into nearby cells. This causes endothelial cells and perivascular astrocytes to swell [38,44,45]. This swelling both compresses capillary lumens from the outside and leads to endothelial blebs protruding into the lumen, both of which impede the flow of perfusate [38,39].

### 3.3. Mural Cell Contraction

Mural cell contraction in ischemia also narrows the microvasculature. In particular, vascular smooth muscle cells, precapillary sphincter cells, and capillary pericytes constrict in response to molecular signals released during ischemia [46,47]. For example, in one mouse study, cardiac arrest caused the average diameter of precapillary sphincters to drop rapidly from approximately 5 μm to 4 μm within minutes, followed by a gradual further constriction to approximately 1.5 μm over the next 25 min, sharply raising the estimated resistance to flow [48]. Mural cell contraction is initially reversible but is thought to become irreversible as ATP depletion locks actin-myosin cross-bridges, in a condition analogous to rigor mortis [49].

### 3.4. Vessel Wall Breakdown

Finally, vessel wall breakdown compromises the ability of the vasculature to contain perfusate. Under normal conditions, the blood–brain barrier (BBB) tightly restricts the movement of fluid and solutes from the vascular lumen into the brain parenchyma. After circulatory arrest, however, the BBB progressively degrades through endothelial cell damage and other mechanisms [50]. As the vessel walls become increasingly permeable, perfusate that enters the vasculature leaks out into the surrounding tissue rather than flowing downstream to perfuse additional regions [12,15]. This extravasation generates vasogenic edema, which further compresses the microvasculature and worsens perfusion impairment. Vessel wall breakdown is arguably the ultimate limiting factor in perfusion of the highly ischemic brain, because beyond a certain degree of degradation, attempting mechanical perfusion simply drives fluid into the parenchyma rather than leading to distribution through the vasculature. The perfusion pressure required to distribute fluid through the body can also become prohibitively high after sufficient degradation [51]. Consistent with this, in embalming, edema is frequently found to occur when perfusion is performed after a long ischemia time, which has been attributed to increased capillary permeability [16].

### 3.5. Summary

Perfusion impairment following ischemia is driven by intravascular obstructions, perivascular cellular edema, mural cell contraction, and vessel wall breakdown. These mechanisms may operate at different levels of the vascular tree. Obstructions in larger vessels could prevent perfusate from reaching entire downstream territories, while capillary-level compression and vessel wall breakdown would be expected to cause more diffuse impairment within regions that do receive some flow. There is considerable uncertainty about the relative contribution of these mechanisms, which likely interact and vary with ischemia duration, temperature, and other experimental conditions. Other factors that may contribute to perfusion impairment but are less well characterized include reduced red blood cell deformability and cold agglutination of red blood cells if the body is cooled [18,52]. Collectively, these mechanisms mean that even modest delays between circulatory arrest and the initiation of perfusion can substantially compromise the delivery of fluid to brain tissue, motivating the interventions reviewed below.

## 4. Anticoagulants

Anticoagulants prevent the formation of clots by interfering with the coagulation process. They include heparin, direct oral anticoagulants (DOACs) such as apixaban, and calcium chelators, including EDTA and citrate, which sequester the calcium ions that are required for multiple steps in coagulation. Historically, heparin has been the most commonly used anticoagulant in perfusion-based brain banking protocols [2,4].

One obvious limitation of anticoagulants is that they can only prevent new clot formation, rather than dissolve clots that have already formed. By the time perfusion is initiated in a typical brain banking workflow, clot formation is expected to have already progressed substantially.

Even setting aside the timing issue, a key question is to what extent anticoagulants would be expected to address the types of intravascular obstructions that actually form during ischemia. Some fibrin formation can (and does seem to) occur in ischemia, as the proteolytic reactions of the coagulation cascade can proceed independently of intact cellular metabolism [27]. However, ischemic coagulation is substantially decreased, because robust thrombin generation requires the assembly of coagulation complexes on activated platelet surfaces [53], and platelets in cadaver blood are generally not activated after death, even in early ischemia [54]. As previously discussed, postmortem clots consist primarily of aggregated red blood cells, which primarily form due to rouleaux formation, with only a relatively small amount of fibrin content [27]. Rouleaux formation is a physical process that depends on the interaction of plasma proteins, especially fibrinogen, with the red blood cell surface [55]. Rouleaux formation is not part of the enzymatic coagulation process and would not be inhibited by anticoagulants, although notably it should also be reversible by mechanical flow [28].

Anticoagulants may be more useful if they are already present in the circulation at or before the time of death, where they could inhibit (a) the formation of agonal thrombi or capillary microthrombi during the dying process and (b) any early ischemic coagulation while platelets and clotting factors are still functional. Consistent with this, some previous literature has found that heparin can partially ameliorate perfusion impairment in the brain when used prior to cardiac arrest [41,42]. In organ transplantation, premortem heparinization is common practice in donation after circulatory death. For example, approximately 92% of donation after circulatory death (DCD) donors in one Canadian cohort received heparin before withdrawal of life-sustaining treatment [56]. However, the evidence that even this well-established clinical practice actually improves transplant outcomes is surprisingly equivocal [56,57].

Taken together, the evidence suggests that anticoagulants are unlikely to be highly effective when added to the perfusate during perfusion of the ischemic brain, but may provide modest benefit when they are present in the circulation before or at the time of cardiac arrest. In brain banking, a substantial fraction of donors may already be on anticoagulants for other clinical indications. Whether such premortem anticoagulant use is associated with improved perfusion quality in brain banking has, to our knowledge, not been systematically studied, and this would be a useful topic for future research.

## 5. Fibrinolytic Agents

Fibrinolytic agents such as tissue plasminogen activator (tPA), urokinase, and streptokinase dissolve blood clots by promoting the generation of plasmin, which degrades fibrin [58]. Because vascular obstructions are thought to be a major contributor to perfusion impairment, fibrinolytics are a logical candidate intervention [24]. However, as previously discussed, postmortem clots differ from antemortem thrombi in that they consist primarily of aggregated red blood cells with relatively modest fibrin content [27], which makes fibrinolytics less likely to be beneficial.

One study in cats found that tPA and heparin administered during CPR after 15 min of cardiac arrest reduced cerebral no-reflow after 30 min of normothermic reperfusion from 28% to 7% of the sectioned forebrain area [43]. However, two factors may limit the translation of these results to brain banking. First, the animals had 30 min of spontaneous recirculation with active cardiac output to distribute these fibrinolytic agents throughout the microvasculature and allow them to act. While extended perfusion times are certainly possible in brain banking, this means that there will be an extended period of autolysis in regions that receive delayed preservation. Second, reperfusion of living tissue with blood triggers secondary coagulation through endothelial activation and platelet recruitment, a process that tPA and heparin would counteract. This mechanism would not be present during perfusion of the ischemic brain with an acellular solution. Notably, tPA activity is substantially decreased at hypothermic temperatures [59], which could also be a factor if the brain was already cooled or cold perfusate were used.

In organ transplantation, previous research has shown that the use of fibrinolytic agents can help to improve microvascular patency [60]. However, to the best of our knowledge, thrombolytics have not yet been incorporated into standard machine perfusion protocols, and instead are an investigational approach.

As with anticoagulants, fibrinolytics may be most relevant in brain banking when they have already been administered premortem for independent clinical reasons. Alternatively, they may also be useful as part of a prolonged warm perfusion protocol in which metabolic recovery is the goal, such as in the BrainEx protocol [61]. In such cases, the extended perfusion times required for thrombolysis to occur are acceptable because the perfusate is designed to sustain metabolism and cellular viability. If fibrinolytics were to be used, they would be most useful if included in the initial perfusate so as to contact intravascular obstructions as early as possible, as their utility depends on having sufficient time for enzymatic action. However, in a brain banking workflow focused on more immediate connectome preservation, the time window for perfusion prior to definitive preservation is limited, which means that there would be less expected benefit from the use of fibrinolytics.

## 6. Vasodilators

Several vasodilators have previously been used in brain banking protocols. For example, sodium nitrite has been included in the washout solution of one protocol [62]. As another example, nimodipine was used as a part of the BrainEx protocol, which was studied on porcine brains that were rapidly flushed 10 min after the cessation of blood flow and then perfused after 4 h of ischemia [61]. In this study, the authors reported that nimodipine led to an increase in cerebral arterial flow velocity, confirming that at least some mural cells retained dilatory responsiveness under those conditions.

Vasodilators such as sodium nitrite can clearly be beneficial in providing vasodilation and enhancing the clearance of blood when administered to living animals with intact vascular function [63]. One study found that three vasodilators, one of which was papaverine, did not lead to a significantly improved perfusion quality in the brains of rabbits after they were first subjected to 15 min of global cerebral ischemia [42]. 

One of the key problems with the use of vasodilators in brain banking is that they require active biomolecular signaling pathways. For example, in the BrainEx study, restoration of cellular metabolism required a complex, oxygenated, hemoglobin-based perfusate with continuous haemodiafiltration [61]. This dependence on active metabolism adds substantial complexity and time to the perfusion protocol prior to definitive preservation. This is discussed further in the Section 18. Alternatively, it is possible that vasodilators could be identified that rapidly improve perfusion quality in ischemic tissue without metabolic restoration, for example via passive mechanisms such as calcium channel blockade. However, it is not clear to what extent vasodilation with such methods is possible, and this would require further investigation.

## 7. Washout Solutions

Washout is the perfusion of a solution through the vasculature before the introduction of the target agent (e.g., fixative or cryoprotectant). The rationale is that flushing blood and associated clots from the vasculature before introducing the preservation solution may achieve more uniform distribution of that solution.

In embalming, this step is known as “pre-injection”. Pre-injection fluids typically contain chemical anticoagulants (e.g., EDTA), surfactants, and other agents designed to dissolve clots, promote capillary flow, and prepare the vascular system for the distribution of arterial fluid [16]. For example, one study described a protocol in which a commercial clot-dispersing solution was perfused at high pressure for 10 to 20 min prior to the introduction of embalming fluid, with simultaneous venous drainage and massage of the extremities designed to flush out blood clots [64]. One source notes that pre-injection fluids were historically more necessary because older arterial embalming formulations were too harsh to allow for blood clearance during fixation itself, and that modern arterial fluid formulations have improved to the point where dedicated pre-injection is not required [16]. However, a historical source from the turn of the 20th century notes that the removal of the blood in embalming prior to the perfusion of fixative-containing solutions was not common even then, and was argued to be unnecessary [65].

In rodent perfusion fixation, a saline or PBS washout step prior to perfusing fixative is very common and is part of most standard protocols [66]. For example, in one protocol used for adult mice, approximately 20 mL of saline is typically perfused, until the outflowing fluid becomes clear of blood, at which point the fixative solution is introduced [67]. However, other sources have noted that a dedicated blood washout is not necessary for achieving high-quality fixation [68,69,70]. When a washout solution is used, its composition should be carefully chosen, as this can affect different properties of the resulting preserved brain, such as the extracellular space [71].

In human brain banking, washout is common but not universal. Slightly more than half of the studies in one review of the literature reported using a washout solution prior to perfusion fixation, with saline or PBS being the most common washout solutions when they were used [2]. Some investigators chose to omit the washout step. Others used washout only in cases with an ischemia time exceeding 12 h, with the reported goal of preventing the fixation of blood clots in place [2,72].

In vascular corrosion casting, some investigators have achieved good microvascular filling without any washout step. In two studies, human brains obtained within 12 h of death were cannulated ex situ at multiple branches of the anterior, middle, and posterior cerebral arteries, and undiluted Mercox resin was injected manually without any prior rinsing of the blood vessels [73,74]. The resulting casts showed detailed capillary-level filling, suggesting that the resin was able to mechanically displace residual blood and traverse the capillary bed.

Collectively, the available evidence suggests that the net effect of a dedicated washout step when using perfusion fixation in brain banking is not well established and may even be negative in some instances [75]. Perfusion of fixative itself can clearly displace blood from the vasculature, because fixation does not occur instantaneously [12]. Specifically, the hydrostatic pressure exerted by the perfusate during the initial seconds of fixative perfusion is far faster than the relatively slow process of chemical crosslinking, which takes minutes [70]. There is therefore no strong a priori reason to expect that the presence of fixative in the perfusate would prevent it from mechanically clearing blood. There is so much blood in the human brain that washing it out can also take a long time (20 min in one study, although this depends on the flow rate), which means it is not a trivial question [4]. A washout step also has significant practical risk, insofar as perfusing the washout solution can cause or worsen tissue edema, particularly in cases with long ischemia times, given that the vessel walls may already be significantly compromised by the time that perfusion begins [12]. If edema develops during the washout phase, the brain may be in a worse state by the time preservative solution is introduced than if perfusion had begun with preservative solution immediately. It may be the case that the use of a washout solution would be useful in some cases or not others. More experimental comparisons between immediate perfusion fixation and washout followed by perfusion fixation would be highly informative.

## 8. Surfactants

Surfactants, also known as “wetting agents” in the embalming literature, are amphiphilic molecules that reduce the surface tension of a liquid. This enables the fluid to spread more easily across surfaces and penetrate into smaller spaces. The use of surfactants has been called one of the major developments in embalming since the introduction of formaldehyde [16]. They are thought to promote distribution through capillaries by reducing the resistance to flow at fluid-tissue interfaces [76,77,78,79]. Surfactants used in embalming formulations include polyethylene glycols and sodium lauryl sulfate [80]. By 1945, pre-injection solutions in embalming, also referred to as “capillary wash” fluids, were reported to sometimes contain surfactants [81].

There are two main ways that surfactants are thought to reduce perfusion impairment [16,80]. First, by reducing the surface tension of the perfusate, they are thought to improve flow through partially occluded or narrowed capillaries. However, to the best of our knowledge, the scientific evidence that these surfactants actually improve capillary-level perfusion under ischemic conditions is limited, and most claims of efficacy derive from empirical reports in the embalming trade literature rather than controlled studies. Second, by reducing the hydrophobic interactions between red blood cell surfaces and otherwise disrupting the cohesive forces within clots, surfactants can help to dissolve and dislodge clots. For example, one study perfused previously embalmed human heads with a solution containing 1.0% detergent (which is a type of surfactant) combined with a proteolytic enzyme, in order to dissolve embalmed blood clots from the vasculature [82]. They found that when they subsequently injected radiopaque media, many of the arterial branches of the carotid artery were patent, including fine vessels supplying the brain. A milder surfactant, poloxamer 188, has been used clinically, where it has been shown to reduce red blood cell aggregation, lower blood viscosity, and improve microvascular blood flow [83].

Surfactants can also be used to deliberately open the BBB, which is relevant in brain banking workflows that require the delivery of relatively larger molecules to the parenchyma via perfusion. SDS (sodium dodecyl sulfate, also known as sodium lauryl sulfate) is a negatively charged surfactant that has been shown to produce dose-dependent increases in BBB permeability in rats [84]. This property was exploited in the development of aldehyde-stabilized cryopreservation, a brain banking method in which brains are perfusion fixed with a glutaraldehyde solution containing SDS to simultaneously fix the tissue and open the BBB, followed by perfusion of the cryoprotectant ethylene glycol [85]. Without BBB opening, perfusion of cryoprotectants causes dehydration, because water exits the brain across the BBB faster than the cryoprotectant enters. SDS-mediated BBB opening allows the cryoprotectant to cross the BBB, thus preventing this osmotic imbalance. This use case is specific to cryoprotectant delivery rather than fixative delivery, because small molecule aldehyde fixatives (such as formaldehyde and glutaraldehyde) cross the BBB readily on their own, and therefore do not require BBB disruption for effective tissue penetration.

Notably, the use of surfactants in brain banking requires careful dosing and consideration of their potential to damage cellular ultrastructure. Ionic detergents such as SDS can solubilize cell membranes by inserting into and disrupting the lipid bilayer. Indeed, ionic detergents such as SDS have been used in tissue decellularization to strip all cellular material and thereby help to create organ scaffolds [86]. In the aldehyde-stabilized cryopreservation protocol, the use of SDS has still been found to be compatible with connectome preservation [87]. This may be in part because the SDS was delivered concurrently with glutaraldehyde fixative, which would help to stabilize cellular structures against any slow-onset SDS-mediated disruption. In protocols where surfactants would be perfused before or without prior fixation, the risk of ultrastructural damage might be greater, possibly benefiting from further research and dose optimization.

## 9. Osmotic Agents and Colloids

**Figure 2:**
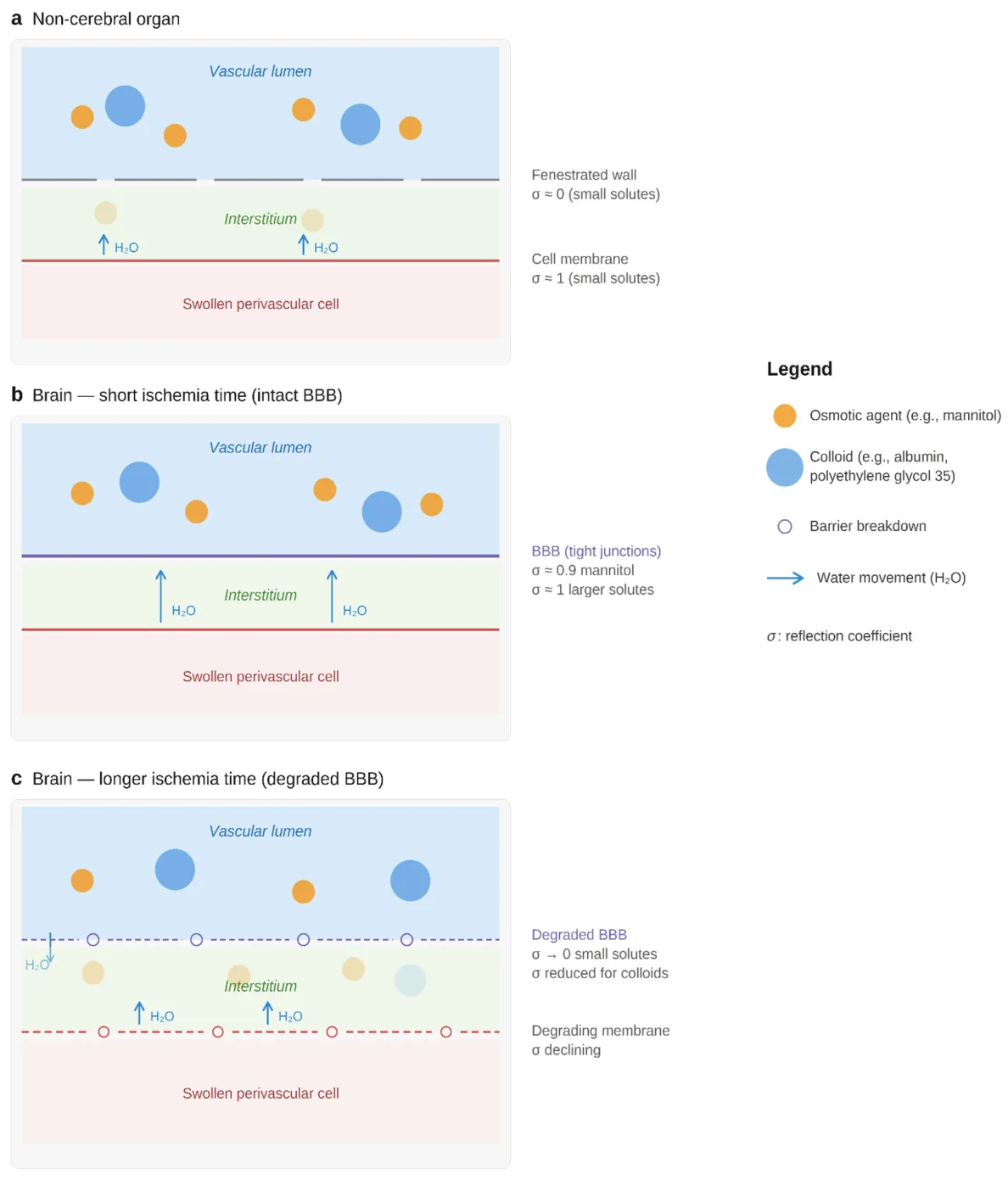
Osmotic agents and colloids act at different biological barriers to improve perfusion. (**a**) In non-cerebral organs, vascular walls have relatively large inter-endothelial cell gaps through which small osmotic agents (e.g., mannitol) pass freely into the interstitium (σ ≈ 0 for small solutes), where they draw water out of swollen perivascular cells across cell membranes (σ ≈ 1 for small solutes). Colloids (e.g., polyethylene glycol 35 kDa) are too large to cross the vascular wall and generate colloid osmotic pressure that retains perfusate intravascularly and opposes hydrostatic filtration into the interstitium. (**b**) In the brain with a short ischemia time and relatively intact BBB, endothelial cells and tight junctions restrict the passage of even small hydrophilic solutes such as mannitol. This causes osmotic agents to behave more like colloids, generating intravascular osmotic pressure. (**c**) At longer ischemia time, ischemia progressively degrades the BBB, lowering the effective reflection coefficient for small solutes (σ → 0), allowing osmotic agents to enter the interstitium and act in their classical role at cell membranes. The reflection coefficient for colloids is also reduced as the BBB degrades, which diminishes their ability to retain fluid intravascularly; higher molecular weight colloids may be more robust against this effect. Cell membranes also degrade with prolonged ischemia, reducing their effective reflection coefficient, and potentially leading to water shifts to the interstitium. BBB: blood–brain barrier; σ: reflection coefficient.

Osmotic agents and colloids address perfusion impairment by creating osmotic pressure gradients at different biological barriers. The distinction between them is based on the reflection coefficient (σ), which quantifies a given membrane’s impermeability to a given solute. The reflection coefficient depends on the ratio of the solute’s molecular radius to the effective pore radius of the membrane [88]. Because cell membranes and vascular walls have different effective pore sizes, the same solute can generate an osmotic pressure at one barrier but not the other (Figure 2). Osmotic agents (also called impermeants in the organ preservation literature) are small molecules that cannot cross cell membranes (σ ≈ 1) but can cross vascular walls (σ ≈ 0), allowing them to enter the interstitial space and draw water out of swollen cells [89]. Colloids (also called oncotic agents) are large macromolecules that cannot cross vascular walls, thus generating a colloid osmotic pressure that retains fluid intravascularly and opposes extravasation.

In the brain, the BBB restricts the para-endothelial cell diffusion of even relatively small hydrophilic molecules. For example, mannitol (182 Da) is largely thought of as an osmotic agent in most organs, but it is thought to be largely excluded by the intact BBB, with an estimated σ of approximately 0.9 as interpolated from its molecular size [89,90,91]. However, even when mannitol is largely excluded from the parenchyma, its intravascular hypertonicity can still reduce brain volume by dehydrating endothelial cells, which in turn draw water from the parenchymal space through a chain of osmotic gradients [92,93]. This is a mechanism exploited in clinical medicine, where mannitol can be used to reduce intracranial pressure during neurosurgery [93]. Sucrose is also considered an osmotic agent in other organs, but not in the brain, where it is actually used as a marker of BBB permeability, as it has very little penetration past an intact BBB [89,94].

A key complication is that the BBB does not remain intact when blood flow ceases. Instead, the BBB is known to degrade progressively during ischemia [95]. How quickly the BBB breaks down during global cerebral ischemia is not very well established, and may differ by the species, temperature, and other factors. This means that a given chemical agent may act as a colloid if it is administered after a minimal amount of ischemia time, but then act as an osmotic agent if given after a substantial amount of ischemia time once the BBB is more degraded. As an example, one study found that the permeability of the BBB to mannitol increased substantially after 10 min of global cerebral ischemia [96]. The BBB also varies regionally, for example not being present in circumventricular organs [97].

A further complication is that osmotic agents can also be used to open up the BBB. An example of this is mannitol itself, as hypertonic mannitol can be used to open the BBB by osmotically shrinking endothelial cells and widening tight junctions [92]. Of particular relevance to brain banking, one study demonstrated that adding 4% (*w*/*v*) mannitol to an aldehyde fixative solution, preceded by a 15% (*w*/*v*) hypertonic mannitol bolus to open the BBB, preserves approximately 20% extracellular space throughout the brain [17]. Compared to standard fixation, which causes a near-total collapse of the extracellular space, mannitol was used in this study to help with tracing connections for connectomics [17].

In organ transplantation, colloids are a standard component of preservation solutions [98]. They are likely increasingly important as the ischemia time increases, as the BBB breaks down further, increasing the risk of vasogenic edema. Several studies have found that the addition of colloids can help with perfusion of the ischemic brain. For example, one study found that the infusion of PEG-20 kDa during CPR in a rat cardiac arrest model improved microcirculatory flow in the brain, which the authors attributed in part to decompression of the microvasculature [99]. In rodent perfusion fixation, adding macromolecules such as polyvinylpyrrolidone (PVP) to the perfused fixative solution has been shown to counteract intravascular hydrostatic pressure and reduce the net outflow of fluid into tissue [100]. Other chemicals that have been used as colloids are hydroxyethyl starch (HES) and dextran 40 [98].

For colloids, an important parameter is the colloid osmotic pressure (COP), which, when it comes to perfusion, generally refers to the pressure that the colloid generates across the vascular wall to retain fluid intravascularly and oppose hydrostatic filtration into the interstitium [101]. Although the osmolality of a typical colloid solution is negligible from the perspective of cell volume regulation, the COP that it generates across the vascular wall can still be substantial. For example, the physiological COP of blood plasma is approximately 25 mmHg, which is generated primarily by albumin and serves as the main counterforce to hydrostatic filtration pressure in the capillaries [101]. In organ preservation solutions, the COP varies depending on the type and concentration of colloid used. For example, the University of Wisconsin (UW) organ preservation solution, which contains 50 g/L of HES, has a COP of approximately 32 mmHg at 5 °C [102]. On the other hand, histidine-tryptophan-ketoglutarate (HTK) solution, which lacks a colloid, has a COP of only approximately 1.5 mmHg [102]. This is despite the two solutions having similar osmolalities, of 310 mOsm/kg H_2_O for HTK and 324 mOsm/kg H_2_O for UW [102]. For long-term perfusion, including substances in the perfusate that exert a COP are considered important to prevent vasogenic edema.

The choice of colloid also affects the viscosity of the perfusate, which is another key consideration during mechanical perfusion, whether following ischemia or not. For example, in UW solution, the high HES concentration (50 g/L) contributes to a viscosity of approximately 5 cP at 5 °C, roughly three times that of HTK [102]. PEG-35 kDa also has a higher viscosity than HES at equivalent concentrations [102]. PEG-35 kDa has been found to generate a higher COP than HES at equivalent concentrations [102]. Higher viscosity increases the vascular resistance during perfusion, which entails a reduced flow at a given perfusion pressure. The tradeoff between increased COP and increased flow resistance that can occur with different types of colloids is one reason that the colloid type and concentration must be carefully considered.

Notably, the COP generated by a colloid depends on how effectively it is retained by the membrane it acts across. In this study, COP was measured using a colloidal oncometer with a semipermeable membrane cutoff of 20,000 Daltons [102]. This means that the COP of a given colloid could potentially be altered depending on how degraded the BBB is during ischemia. Colloids with higher molecular weight, such as high molecular weight versions of HES (450–480 kDa), and Dextran 500, may be particularly robust against the loss of effective COP as the BBB degrades [103].

The required COP of the perfusate is also related to the perfusion pressure used. According to the Starling equation, the COP of the perfusate must counterbalance the net hydrostatic filtration pressure to prevent excessive fluid extravasation. At a lower perfusion pressure, the required COP will be correspondingly lower. For example, for an artificial perfusion protocol with a perfusion pressure at 25% of physiological pressure, one study used the Starling equation to calculate that the target COP to maintain a near-zero net filtration pressure would be approximately 15 mmHg [102]. One reason this is relevant to brain banking is because if a higher perfusion pressure is used to help overcome perfusion impairment in the ischemic brain, then a corresponding higher COP would be required to prevent the increased hydrostatic pressure from driving excessive fluid extravasation into the parenchyma.

Osmotic agents and colloids may be complementary in addressing perfusion impairment. Osmotic agents can help to address capillary compression by shrinking swollen perivascular cells, while colloids counteract hydrostatic filtration and thereby allow higher perfusion pressures with a lower risk of causing vasogenic edema. One study found that a small volume intravenous infusion of both hypertonic 7.5% NaCl and 6% HES during CPR after 15 min of cardiac arrest in cats significantly reduced cerebral no-reflow [104].

In practice, the effects of osmotic agents and colloids are likely to depend strongly on the duration of ischemia and the degree of vascular barrier disruption. The highest efficacy might therefore be achieved if the agents that are used (if any), and their concentration, are adjusted to the condition of the individual brain, rather than being applied as a fixed formulation.

## 10. Perfusion Pressure

Higher perfusion pressures can address nearly all of the major mechanisms of perfusion impairment (with the exception of vessel breakdown, which is discussed below). Consistent with this, one source has argued that increasing perfusion pressure is the single most important intervention for no-reflow [40]. In support of this, they cited one study in which raising the perfusion pressure consistently ameliorated no-reflow in rabbits after up to 30 min of ischemia [105]. In addition, a resuscitation study found that the controlled reperfusion at carotid pressures above 50 mmHg achieved complete neurological recovery in pigs after 30 min of normothermic global brain ischemia. On the other hand, the same perfusate delivered at lower pressures resulted in the infarction of deep and watershed regions, suggestive of inadequate perfusate distribution [106]. 

Notably, in perfusion fixation, flow rate is typically controlled by a peristaltic pump, while pressure is the observed dependent variable. The observed perfusion pressure at a given flow rate is therefore a measure of the resistance of the vasculature (R = P/Q).

In rodent perfusion fixation with no ischemia, gravity-based systems often use perfusion pressures of approximately 50 to 75 mmHg [66]. However, other investigators have instead used higher perfusion pressures and reported that they achieved high-quality histological results. One study of rat perfusion fixation recommended perfusion pressures at or above the typical systolic pressure (~147 mmHg in their protocol) to ensure rapid fixative delivery, although they did not find significant ultrastructural differences between the use of ~81 and ~147 mmHg on their EM evaluation [100]. In this study, higher perfusion pressure did also cause greater parenchymal volume and associated ventricular narrowing, consistent with more fluid being delivered across the capillary walls into the parenchyma [100]. Another source used a pressure of 30 kPa (225 mmHg) for rodent perfusion fixation in order to achieve high-quality structural preservation [23].

One study compared perfusion pressures between 50 and 300 mmHg in mice with no ischemia prior to perfusion [107]. They found that the lower pressure of 50 mmHg was clearly inferior, leading to more dark neurons, perivascular rarefaction, and retained intravascular material. At pressures of 100 mmHg and above, they found that parenchymal preservation was broadly similar. However, they reported that a perfusion pressure of 300 mmHg caused significant BBB disruption and vessel dilation. Their finding that high perfusion pressure can cause BBB disruption is corroborated by previous literature [108]. This will likely also interact with the effects of osmotic agents and colloids. Notably, in these rodent perfusion fixation studies, even though there is effectively no ischemia time, there still could be an analogous degree of perfusion impairment present.

Perfusion pressures used in embalming and anatomical preparation are often substantially higher than those used in neuroscience research, which is likely related to the different goals of the procedure as well as the typical degree of ischemia time. One embalming textbook describes moderate perfusion rates as corresponding to pressures of approximately 2–10 PSI (103–517 mmHg) [16]. One source used an even higher pressure of 30 PSI (1551 mmHg) to perfuse a clot-dispersing solution [64]. Another source used a pressure of 10–20 PSI (517–1034 mmHg) for distributing embalming fluid for anatomical preservation [109].

On the other hand, some anatomical preservation protocols use “gentler” approaches. In one protocol, embalming fluid is delivered either by gravity through carboys secured at 6 feet (134 mmHg) or by an embalming machine set at 2 PSI (103 mmHg) initially at the slowest infusion rate possible, using 28–38 L to perfuse the tissues of the body [78]. One historical source describes an embalming protocol for medical school dissection using gravity-driven hydrostatic pressure with an irrigator stand raised 18–24 inches above the body (approximately 34–45 mmHg), with the body left in this position for approximately 24 h [65].

Although higher perfusion pressure can help overcome perfusion impairment due to clots, edema, and mural cell contraction, it is not a panacea. Higher pressure increases the risk of vasogenic edema and vessel rupture. One source notes that it is preferable to inject fluid with a low pressure to avoid the risk of vessel rupture, which, when it occurs, can be detected on the basis of a sudden increase in flow and/or decrease in pressure [65]. The severity of this tradeoff is expected to worsen with increasing ischemia time, as the integrity of the vessel wall progressively degrades, even as other aspects promoting perfusion impairment worsen.

Notably, the optimal perfusion pressure is not a fixed value, but depends on the vascular resistance of the specific specimen being perfused. Vascular resistance will vary substantially based on factors such as the underlying vascular anatomy, the degree of pre-existing vascular pathology, the duration of the agonal state, and the ischemia time. As a result, the same perfusion pressure can produce very different flow rates in different brains, and vice versa. Consistent with this, we have previously found that achieving the same flow rate in donated human brains requires higher perfusion pressures in brains with longer ischemia times, which is likely due to the increased vascular resistance that accumulates with ischemia time [12]. What matters for perfusion quality is the pressure observed at a given flow rate, i.e., the flow rate-pressure relationship, rather than either variable in isolation. This means that targeting a specific pressure without also considering flow may result in either inadequate perfusion (if vascular resistance is high) or excessive extravasation (if vascular resistance is low). However, this aggregate measurement can also mask spatial heterogeneity in vascular resistance. The mechanisms leading to perfusion impairment act locally and heterogeneously across the vascular tree. This means that there is the potential for preferential flow through regions of lower resistance and inadequate fixative delivery to regions with higher local vascular resistance, even when the aggregate flow and pressure appear adequate.

Using pulsatile flow, which may provide benefits in cardiopulmonary bypass surgery [110], may also be a method for improving perfusion quality without increasing the average perfusion pressure. The intermittent pressure peaks generated by pulsatile flow would produce greater shear forces at the fluid-vessel wall interface, which is one of the factors known to be capable of dispersing rouleaux-based clots [55]. At the same time, it would potentially lead to less sustained extravasation through degraded vessel walls, and thus vasogenic edema, than an equivalent constant pressure would. Notably, pulsatile perfusion was one of several features of the OrganEx system that achieved improved organ perfusion in pigs after one hour of warm ischemia [111]. It is also a component of a brain resuscitation method that led to consistent neurological recovery after 30 min of global brain ischemia in pigs, whereas non-pulsatile delivery at the same flow rate yielded variable outcomes [112]. Pulsatile perfusion is a promising strategy that should be compared experimentally with continuous flow at matched mean pressures and flow rates, using regional perfusion quality and ultrastructural preservation as outcome measures.

On the opposite side of high-pressure perfusion, one alternative strategy is prolonged but low-pressure perfusion. Even if capillary perfusion is not fully restored, aldehyde fixatives are small molecules that can diffuse from partially perfused vessels into the surrounding parenchyma. Combined with a high molecular weight colloid, this method could in principle allow gradual fixative delivery to poorly perfused regions while minimizing the risk of vasogenic edema. However, such low pressures would be unlikely to displace intravascular clots or overcome significant mechanical obstructions, which means that some areas of the brain may still not be well perfused. The effectiveness of a low perfusion pressure approach has not been experimentally evaluated, but it warrants evaluation, especially in cases where long periods of ischemia have compromised vessel wall integrity to the point that higher perfusion pressures would be expected to cause severe vasogenic edema.

In practical terms, the optimal perfusion pressure which bests balances the goals of improving distribution and avoiding extravasation or structural distortion will almost certainly vary among brain specimens. In addition, perfusion pressure would ideally be evaluated alongside the flow rate and the regional perfusion quality, rather than being targeted as an isolated measure on its own.

## 11. Hypothermia

Cold temperature is clearly one of the most powerful known methods for ameliorating the accumulation of perfusion impairment, although the relationship is complex. On one hand, hypothermia slows the metabolic processes that drive several of the mechanisms discussed above, which slow the development of perfusion impairment [61]. Hypothermia also slows autolysis, thus improving the ultrastructure preservation of the tissue [45]. On the other hand, cold temperature can also promote edema during perfusion. The net effect likely depends on the depth of cooling, its timing relative to the initiation of ischemia, and the method used for perfusion.

Several lines of evidence suggest that hypothermia can ameliorate perfusion impairment, especially when it is initiated prior to ischemia, as has been routinely used in deep hypothermic circulatory arrest [113]. For example, in one study, cooling to 28 °C before and during cardiac arrest eliminated the detrimental effect of a prolonged no flow interval on cerebral blood flow during subsequent CPR [114]. At normothermia, extending the duration of no flow from 1.5 to 6 min reduced forebrain blood flow during CPR from approximately 18.6 to 6.1 mL/100 g per minute, whereas at 28 °C the blood flow during CPR was largely maintained despite the same increase in the duration of no flow [114]. The protective effect appears to act specifically on reflow, since the authors noted that hypothermia to this degree otherwise raises cerebral vascular resistance and lowers baseline blood flow. The authors propose that one potential mechanism is that hypothermia could reduce the ischemia-driven cell swelling that contributes to capillary compression.

Mild hypothermia also seems to act on the microvasculature somewhat independently of its effect on tissue necrosis. In a rabbit model of myocardial infarction, topical cooling of the heart to approximately 32 °C reduced the spatial extent of no-reflow by 43% when initiated 5 min before reperfusion and by 38% when initiated 5 min after, even though the infarct size was not significantly reduced [115]. Remarkably, even when cooling was delayed until 30 min after reperfusion, it still reduced the spatial extent of no-reflow by 30% [115]. The authors interpreted this as evidence that microvascular damage is largely due to reperfusion injury, and that the microvasculature is particularly receptive to the protective effects of cooling. Whether an analogous benefit would occur in the brain during acellular perfusion, wherein there is no reperfusion injury in the typical sense, is unclear.

Cold temperature also has potential risks for perfusion quality. In the organ preservation and critical care literature, exposure of tissues to cold has been found to increase vascular permeability and promote edema [116,117]. In the brain specifically, perfusion at deep hypothermic temperatures (1 °C to 4 °C) has been found to lead to the progressive development of edema [118]. The cold preservation literature also shows that perfusion of certain perfusates, such as saline, at cold temperatures can be directly harmful to cells. For example, one study found that flushing rat kidneys exposed to 30 min of ischemia with ice-cold saline prior to reperfusion caused more severe tubular necrosis than the same duration of ischemia followed by reperfusion alone at body temperature [119]. The authors propose that cold exposure leads to structural changes in membrane lipid bilayers while anoxia drives Na+/K+ pump dysfunction, and that together these lead to cytoskeletal disruption and cell edema [119].

The practical implication for brain banking is that although hypothermia is remarkably protective against perfusion impairment, if the brain is perfused at cold temperature, then edema may be a particular concern. This could narrow the window between the perfusion pressure needed to overcome perfusion impairment and the pressure that causes excessive extravasation. One way to manage this tradeoff would be to target lower perfusion pressures at colder temperatures.

## 12. Lipophilic Perfusates

In postmortem angiography, lipophilic perfusates have been used to address the vessel wall breakdown that occurs after prolonged ischemia. In this two-step approach, the vascular system is first perfused with an oily liquid (such as diesel oil or paraffin oil diluted with 20% decane), and then a lipophilic contrast agent is injected as a bolus for radiographic imaging [15,120]. Lipophilic solutions are immiscible with the aqueous interstitial fluid, so they remain intravascular despite the increased permeability of the ischemic vessel wall. The oily solutions have also been reported to help in flushing out intravascular clots [15], although this may simply be due to flow of fluid through vessels. However, the oily perfusate occludes small arterioles, blocking subsequent aqueous perfusion at vessel diameters of 50 μm or less [120]. The authors speculated that the oily perfusate could enter the venous system via arteriovenous shunts rather than traveling through the capillary networks [120]. However, the existence of physiological cerebral arteriovenous shunts is debated, with the available evidence suggesting they are sparse in the normal human brain [121]. Regardless of the mechanism, the result is that aqueous fluid that is perfused after the oil becomes arrested at the oil-occluded arterioles. This means that if an oily perfusate were to be used to help with removing intravascular obstructions, it would need to be removed via perfusion with other substances in order to allow for subsequent capillary perfusion.

## 13. Retrograde Venous Perfusion

In retrograde venous perfusion, fluid is delivered through cannulation of the venous system rather than the arterial system. Filling the cerebral venous system with colored silicone or latex via cannulation of the internal jugular veins is commonly performed in neurosurgical education applications [122,123]. In these protocols, the internal jugular veins are cannulated in the mid-neck, above the valves that have been identified as a major barrier to retrograde cerebral perfusion via the superior vena cava in the cardiac surgery literature [124,125]. However, in these applications, the goal is to fill the venous compartment itself rather than to deliver fluid through the capillary bed to the parenchyma. The superficial cerebral veins and dural sinuses are highly anastomosed [126], which may lead perfusate to primarily distribute through these large, low-resistance venous channels rather than entering the smaller parenchymal venules. Whether retrograde venous perfusion could achieve meaningful capillary-level delivery is uncertain, although capillary-level delivery may not be necessary if the goal is fixative diffusion rather than washout or oxygen delivery. Theoretically, venous perfusion at low pressure could be used to fill the venous compartment with a fixative solution that would then diffuse into the surrounding parenchyma over time, complementing the arterial perfusion of regions that are poorly reached from the arterial side. However, this approach has, to our knowledge, not yet been evaluated in the context of perfusion fixation.

## 14. Decompressive Craniectomy

Decompressive craniectomy addresses perfusion impairment by relieving the external tissue pressure that develops in the swollen brain. In malignant hemispheric infarction, removal of a large bone flap converts the cranium from a closed compartment into a compliant one, lowering intracranial pressure and increasing the transmural pressure available to keep vessels open. This addresses any perfusion impairment caused by macroscopic tissue swelling and associated pressure-mediated vascular collapse, but not from intravascular obstruction. Patients requiring decompressive craniectomy after malignant hemispheric infarction exhibit marked impairment of the cortical microcirculation, with intermittent or absent flow in many microvessels compared with controls [127]. Imaging studies performed before and after decompression have found improved cerebral hemodynamics after decompressive craniectomy, including increases in cerebral blood flow across the measured territories and increases in cerebral blood volume in penumbral tissue [128,129].

In the brain banking literature, decompressive craniectomy has not, to our knowledge, been evaluated as a strategy to improve perfusion. It is obviously only relevant in contexts where the brain has not already been removed from the skull, which are a minority of brain banking workflows [2,130]. Theoretically, if perfusion is performed in situ with a closed cranium, decompressive craniectomy could be helpful, particularly in cases with severe hemispheric swelling. However, experimental evidence for such a benefit is lacking. Moreover, decompressive craniectomy might introduce an additional procedural step before the tissue is actually preserved, and any potential benefits would therefore need to be balanced against its risks.

## 15. Sonothrombolysis

Sonothrombolysis refers to the use of ultrasound, often combined with microbubbles, to enhance clot dissolution. Proposed mechanisms include acoustic cavitation, microstreaming, radiation forces, and improved penetration of fibrinolytic agents. In acute ischemic stroke, early clinical studies suggested a biological effect, with the original CLOTBUST trial finding higher rates of early complete recanalization when 2-MHz transcranial ultrasound was added to intravenous tPA. However, later trials have been more mixed. The phase 3 CLOTBUST-ER study found the approach feasible and likely safe, but did not demonstrate a clear improvement in functional outcome, while meta-analyses have generally concluded that sonothrombolysis improves recanalization without a clear increase in symptomatic intracranial hemorrhage or death [131,132,133,134].

In a rat stroke model, microbubble-enhanced sonothrombolysis restored microvascular vascular volume in the affected hemisphere, whereas rt-PA alone did not, supporting the idea that ultrasound plus microbubbles may help reverse no-reflow at the level of the microcirculation [135]. This is a conceptually attractive approach for brain banking, but to the best of our knowledge, is hitherto untested.

## 16. Summary

We have attempted to review the major classes of interventions that have been used to ameliorate perfusion impairment that might be relevant in brain banking (Table 1). For example, anticoagulants and fibrinolytics appear to be partially helpful when administered prior to cardiac arrest, but have not been found to offer clear benefit when they are added to the perfusate after ischemia has already initiated. Vasodilators have shown mixed results and generally seem to require active cellular metabolism to function, which limits their applicability in ischemic tissue, in the absence of an extended perfusion designed to maintain cellular viability. Osmotic agents and colloids address the major mechanisms of perfusion impairment, and already have well established use in organ preservation, making them promising additions to perfusate solutions, at least at reasonable doses. Perfusion pressure optimization appears potentially very beneficial, though it must be balanced against the downside risks, including vasogenic edema and vessel rupture, particularly at longer ischemia times. Hypothermia is among the most powerful interventions, but cold can itself promote edema during perfusion. Because the mechanisms of perfusion impairment are multifaceted, a combination of these approaches, tailored to the specific specimen and perfusion system, is most likely to yield the best results.

## 17. Limitations of This Review

There are several limitations of this review. First, the evidence reviewed here is highly heterogeneous. For example, the studies reviewed can differ in species, duration of ischemia, temperature, composition of the solution perfused, and whether the circulation was restored with blood or an acellular solution. They also use different outcomes to measure perfusion quality, such as vascular filling, regional blood flow measurements, neurological recovery, red blood cell clearance as seen on histology, or structural preservation quality. An intervention that improves one of these outcomes does not necessarily improve the others.

Second, the experimental evidence of the effects of interventions following global cerebral ischemia in human brains is particularly limited. Much of the available evidence comes from animal models, other transplanted organs, resuscitation experiments, postmortem angiography, or embalming practice. The goals of these experimental settings can differ substantially from the goals of human brain banking intended to preserve tissue ultrastructure. As a result, evidence that a given intervention improves vascular flow or organ function in one setting does not necessarily mean that it would improve the whole brain delivery of preservative chemicals in a brain banking setting.

Third, there is a lack of standardized experimental models and outcome measures across studies. Differences in ischemia duration, perfusion conditions, and methods used to assess perfusion quality make it difficult to compare interventions directly. More standardized animal models that are known to be translatable to ischemic human brains would be helpful. In addition, more comprehensive assessment of perfusion quality across the whole brain is needed, because measurements from a limited number of regions may fail to detect spatially heterogeneous perfusion impairment. Studies should also evaluate both histological and ultrastructural preservation, since improved vascular filling does not necessarily indicate improved preservation of the cellular structures relevant to connectomics.

Fourth, the practical applicability of the reviewed interventions varies considerably. Some interventions could be easily added to an existing rapid perfusion fixation workflow in a brain bank, such as modifying the perfusion pressure or adding an osmotic agent, colloid, or surfactant to the perfused solution. On the other hand, other interventions would require more complex equipment, prolonged perfusion durations for hours, or restoration of active circulation. Their feasibility may therefore depend on factors such as protocol complexity, cost, technical expertise, regulatory requirements, and ethical considerations.

## 18. Future Directions

One key open question is to what extent higher perfusion pressures cause damage to cellular structures in the brain parenchyma. One study found that a perfusion pressure of 300 mmHg caused significant vessel dilation and BBB disruption (as indicated by Evans blue extravasation), while pressures of 125 to 150 mmHg maintained vascular integrity [107]. On the other hand, increasing perfusion pressure up to approximately 150 mmHg substantially reduced parenchymal artifacts such as dark neurons and perivascular spaces, with no clear additional benefit or damage observed in the parenchyma at 300 mmHg. For brain banking with the goal of connectome preservation, vasculature structure is not the primary target of preservation. However, that study used light microscopy, and it is unclear whether high perfusion pressures could damage cellular ultrastructure in ways that would not be detected at that resolution. Moreover, some of the perfusion approaches discussed in this review use pressures far exceeding 300 mmHg, which may help overcome perfusion impairment but could also worsen cellular preservation in ways that have not yet, to the best of our knowledge, been characterized. This uncertainty is particularly relevant because, without the autoregulatory mechanisms present in vivo, artificial perfusion cannot ensure a uniform pressure distribution across the vascular tree, potentially necessitating higher pressures to reach all of the brain regions. Methods to monitor regional flow during perfusion, rather than relying solely on aggregate flow and pressure measurements, would help to characterize this heterogeneity and could eventually lead to the development of more adaptive perfusion protocols.

Future studies could potentially even combine spatially resolved brain imaging measurements with continuous flow–pressure data to develop adaptive perfusion protocols in which the pressure, flow, or perfusate composition is adjusted according to the response of the individual specimen. As larger such datasets become available, computational or machine learning methods could potentially assist in identifying these response patterns and optimizing protocol adjustments.

A complementary paradigm to immediately initiating blood washout and/or preservative perfusion is to attempt to restore blood circulation and metabolism prior to perfusion with preservative solutions. This paradigm has been most developed in the field of medical biostasis, particularly in protocols for attended cardiac arrest where the goal is to maintain brain viability during the transition to cryoprotectant perfusion, but it also has potential relevance to brain banking for connectomics [136,137,138]. In this approach, cardiopulmonary support is initiated after circulatory arrest to attempt to restore oxygenated blood flow, alongside the induction of hypothermia to slow metabolic demand. At the same time, protective medications are administered, including anticoagulants, pH buffers, fibrinolytics, vasodilators, neuroprotective agents, and anesthetics to prevent any return of awareness [136,137]. The primary rationale for restoring circulation is to make the time required to establish vascular access for blood washout less damaging compared to unmitigated warm ischemia, similar to how providing CPR during any unavoidable delay before defibrillation improves neurological outcomes in witnessed cardiac arrests compared with no CPR during that interval [139]. Secondarily, by partially restoring cellular metabolism and blood flow before washout, this approach may allow pharmacological interventions such as vasodilators and fibrinolytics to be more effective. This is because these agents generally require active enzymatic or signaling processes to function. The BrainEx and OrganEx systems have demonstrated that targeted perfusion with cytoprotective solutions can restore aspects of cellular and molecular processes in porcine brains and whole bodies after up to one hour of warm ischemia [61,111]. A key tradeoff is that restoring metabolism introduces the risk of reperfusion injury (depending upon the duration of ischemia) and extends the interval before definitive preservation, during which ongoing autolytic processes may compromise brain structure in regions that are not yet well perfused. To the best of our knowledge, the efficacy of this approach for improving perfusion quality and subsequent preservation of brain ultrastructure has not yet been thoroughly evaluated, and the conditions under which its potential benefits outweigh its risks are uncertain.

Given that the severity of perfusion impairment increases with ischemia time, one of the most obvious strategies for improving perfusion quality in brain banking is to minimize the interval between circulatory arrest and the initiation of preservation. Rapid autopsy programs have been developed in several fields, particularly in oncology, where the goal is to procure fresh tumor tissue for molecular analysis [140]. In the neuroscience context, the one Alzheimer’s Disease Research Center reported performing 51 brain autopsies with postmortem intervals of less than one hour [141]. Other rapid autopsy programs for brain tissue procurement have achieved average postmortem intervals of several hours or less [142,143]. The operational frameworks of these programs, including advance consent, 24/7 on-call staffing, and close coordination with clinical teams and families, provide a model for how brain banking for connectomics could be organized to minimize ischemia time. However, such rapid donation programs are logistically complex and resource-intensive, and they require sustained institutional commitment, which is of course only available in certain settings.

## 19. Conclusions

Perfusion impairment is one of the central unsolved problems in preparing ischemic brain tissue for research. It is likely that there are limits to how well it can be done in certain circumstances, even with ideal methods, but these limits are not yet well defined. Notably, most of the interventions discussed in this review would be experimental if used in the context of human brain banking and will require prospective validation before routine use. Minimizing ischemia time and optimizing the perfusion pressure while monitoring for edema currently have the strongest rationale, whereas many of the pharmacological and mechanical interventions discussed are supported primarily by indirect evidence alone. The development of real-time perfusion monitoring, such as flow–pressure relationship tracking or the ability to monitor flow through particular parts of the brain tissue during perfusion, could potentially allow for interventions to be tailored to the spatial vascular resistance profile of each specimen. However, it is important to remember that in brain banking, improved perfusion is a means to an end, not an end in itself. The goal of brain banking for connectomics is to preserve the molecular and structural features that encode synaptic connectivity. Many of the interventions reviewed here carry their own risks to cellular architecture, whether through osmotic stress, membrane disruption, or mechanical forces from high perfusion pressures. An intervention that improves vascular distribution but damages the structures it is meant to help preserve may be counterproductive. More broadly, as connectome imaging technologies continue to advance, the quality of tissue preparation may become more of a bottleneck. Therefore, progress on ameliorating perfusion impairment may be a key component for scaling human connectomics and could also become a source of insights for clinical fields addressing the same aspects of vascular pathophysiology.

## Figures and Tables

**Table 1:** Summary of interventions for perfusion impairment in the ischemic brain. The “Principal evidence basis” column indicates the main setting(s) from which the experimental support for each intervention is primarily derived.

Intervention Class	Primary Mechanism(s) Addressed	Timing of Use	Expected Utility for Improving Perfusion	Principal Evidence Basis	Key Limitations
Anticoagulants	Intravascular obstructions (fibrin formation)	Most effective prior to cardiac arrest	Low if only used post-cardiac arrest; moderate if used pre-cardiac arrest	Animal brain studies; clinical organ donation evidence	Cannot dissolve existing clots; postmortem clots are primarily RBC aggregates, and are not fibrin-rich
Fibrinolytics	Intravascular obstructions (fibrin degradation)	Most effective prior to cardiac arrest or with extended perfusion	Low in standard workflows if used post-cardiac arrest; may be moderate in extended perfusion protocols	Animal brain studies; experimental organ perfusion evidence	Require time to act; reduced activity at hypothermic temperatures; postmortem clots have limited fibrin
Vasodilators	Mural cell contraction	During perfusion	Uncertain, but current agents are expected to be low	Animal brain studies; metabolically supportive porcine brain perfusion	Require active cellular signaling; many do not address mural cell contraction per se
Washout solutions	Intravascular obstructions (mechanical displacement)	Before fixative perfusion	Uncertain	Rodent fixation studies; brain banking practice	Risk of worsening edema; delays fixation; net benefit not established
Surfactants	Intravascular obstructions (clot dispersal); capillary flow resistance	During perfusion	Uncertain; potentially moderate (at low concentrations)	Embalming practice; limited experimental and clinical evidence	Risk of membrane damage, especially without prior fixation; dose optimization needed
Osmotic agents	Perivascular cellular edema	During perfusion	High	Animal brain studies; clinical and experimental organ preservation evidence	BBB status affects mechanism of action; may cause osmotic shock
Colloids	Vessel wall breakdown (excessive fluid extravasation)	During perfusion	High	Animal brain and resuscitation studies; organ preservation evidence	Increasingly important but also less effective as vessel walls degrade; may cause osmotic shock
Perfusion pressure optimization	All mechanical obstructions (clots, edema, mural cell contraction)	During perfusion	High	Animal brain studies; resuscitation and embalming practice	Higher pressure increases risk of vasogenic edema and vessel rupture; optimal pressure varies by specimen
Hypothermia	All mechanisms (slows metabolic processes driving impairment)	Best initiated before ischemia	High if started pre-cardiac arrest or if there is an unavoidable delay between cardiac arrest and perfusion	Animal brain and cardiac studies; clinical resuscitation principles	Cold can increase vascular permeability during perfusion, increasing the risk of vasogenic edema
Lipophilic perfusates	Vessel wall breakdown (excessive extravasation)	During perfusion	Low	Human postmortem angiography studies	Occludes arterioles, blocking subsequent aqueous perfusion
Retrograde venous perfusion	Reaches regions poorly perfused from the arterial side	During perfusion	Uncertain	Anatomical preparation studies; mechanistic inference	May distribute through large venous channels rather than capillaries; largely untested for perfusion fixation
Decompressive craniectomy	Macroscopic swelling and pressure-mediated vascular collapse	Before (or potentially during) closed-cranium perfusion	Low	Clinical stroke studies; mechanistic inference for brain banking	Takes time during which impairment may worsen; only relevant if perfusion is done with an intact cranium
Sonothrombo-lysis	Intravascular obstructions (clot dissolution)	During perfusion	Uncertain, but potentially high with more research and development	Animal stroke and clinical stroke studies; untested in brain banking	Untested in brain banking; mixed clinical efficacy data

## Data Availability

No new data were created or analyzed in this study. Data sharing is not applicable to this article.

## Abbreviations

BBB	Blood–brain barrier
EM	Electron microscopy
CPR	Cardiopulmonary resuscitation
tPA	Tissue plasminogen activator
DCD	Donation after circulatory death
SDS	Sodium dodecyl sulfate
PVP	Polyvinylpyrrolidone
COP	Colloid osmotic pressure
UW	University of Wisconsin
HES	Hydroxyethyl starch
HTK	Histidine-tryptophan-ketoglutarate
PEG	Polyethylene glycol
DOAC	Direct oral anticoagulant
EDTA	Ethylenediaminetetraacetic acid
PBS	Phosphate-buffered saline
